# Mercy Hospital

**Published:** 1870-03

**Authors:** 


SERVICE OF PROF. BYFORD, THURSDAY, FEB. 3, 1870.

---

VESICO-VAGINAL FISTULA.—Prof. W. H. Byford, after explaining the different kinds of fistulæ, called the attention of the class to Mrs. M. A., aged 23 years, from Wisconsin. She was married four years ago, and was confined about two years since. A midwife was in attendance, and allowed the child's head to remain three days impacted in the pelvis, before she would allow a physician to be called. The physician called was unable to remove the child by forceps; and, on consultation, craniotomy was resorted to. The impaction of the child's head caused a slough between the bladder and vagina, about two inches transversely by three-quarters in the centre; since which time the water has flowed continually through the vagina. The patient, having been etherized, was placed in position for the operation. This consisted in placing her on her side, hips elevated by pillows, knees drawn up, with head and shoulders bent forward. Sim's speculum was now introduced, and confided to the hands of an assistant, while another kept the patient sufficiently etherized. Each member of the class was shown the fistula, and their individual attention called to each subsequent part of the operation. All the instruments used have long handles. The Prof. proceeded to pare carefully the edges of the fistula, with the tenaculum and short-bladed and curved scissors. Two small sponges, in holders, were kept constantly washed, ready to mop up any blood, which was very little.

Nine interrupted silver wire sutures, about a quarter of an inch apart, and nearly the same distance from the free edge, were then introduced. A short, curved needle, armed with a silk thread ligature was used. It was introduced through the lips of the wound, by means of the porte-needle forceps, and drawn through; then the silver wire was attached to the thread and drawn through. These were held out of the way. The Prof. now took the two ends of each silver thread in the wire twister, and adjusted the lips of the wound, by means of the blunt-hook; then, with the aid of the adjustor, the wires were twisted until the lips of the wound were sufficiently in contact. Each suture was cut off about an half inch from the surface and bent over. The operation being now concluded, the patient was carried to bed and laid upon her side. A catheter was introduced into the bladder, to which a piece of rubber tubing had been previously attached. Through this, the urine trickled into a vessel for the purpose. An anodyne of 40 drops of laudanum had been given previous to the operation. No other anodynes were required. Tinct. ferri. chlor., in 15-drop doses, were given four times daily. A week after the operation, the wound looked well, no leakage having occurred. I may add, this is the sixth case operated on by the Prof. before the class.

